# Towards the Better: Intrinsic Property Amelioration in Bulk Metallic Glasses

**DOI:** 10.1038/srep27271

**Published:** 2016-06-08

**Authors:** Baran Sarac, Long Zhang, Konrad Kosiba, Simon Pauly, Mihai Stoica, Jürgen Eckert

**Affiliations:** 1 IFW Dresden, Institute for Complex Materials, Helmholtzstr. 20, D-01069 Dresden, Germany; 2 Shenyang National Laboratory for Materials Science, Institute of Metal Research, Chinese Academy of Sciences, 110016 Shenyang, China; 3 Politehnica University of Timisoara, P-ta Victoriei 2, RO-300006 Timisoara, Romania; 4 Erich Schmid Institute of Materials Science, Austrian Academy of Sciences (ÖAW), Jahnstrasse 12, A-8700 Leoben, Austria; 5 Department Materials Physics, Montanuniversität Leoben, Jahnstrasse 12, A-8700 Leoben, Austria

## Abstract

Tailoring the intrinsic length-scale effects in bulk metallic glasses (BMGs) via post-heat treatment necessitates a systematic analyzing strategy. Although various achievements were made in the past years to structurally enhance the properties of different BMG alloys, the influence of short-term sub-glass transition annealing on the relaxation kinetics is still not fully covered. Here, we aim for unraveling the connection between the physical, (thermo)mechanical and structural changes as a function of selected pre-annealing temperatures and time scales with an in-house developed Cu_46_Zr_44_Al_8_Hf_2_ based BMG alloy. The controlled formation of nanocrystals below 50 nm with homogenous distribution inside the matrix phase via thermal treatment increase the material’s resistance to strain softening by almost an order of magnitude. The present work determines the design aspects of metallic glasses with enhanced mechanical properties via nanostructural modifications, while postulating a counter-argument to the intrinsic property degradation accounted for long-term annealing.

The today’s tendency for creating high performance materials is towards generating advanced alloys with controllable properties^1^,^2^,^3^. Bulk metallic glasses (BMGs) are within this category due to their high thermodynamical metastability when quenched at sufficiently high cooling rates from the liquid state, where the precipitation of the second phase(s) can be tuned by the selected processing method^4^,^5^. Among metallic glasses, multicomponent CuZr-based alloys possess excellent glass-forming ability (cooling rates as low as 1–10 K/s) in combination with high strength (up to 2.5 GPa)^6^, a large elastic limit of 2%, and decent fracture toughness (as high as 100 MPa m^−1/2^)^7^. As opposed to conventional metallic alloys such as steels, due to the lack of grain boundaries, these materials exhibit strain softening behaviour at room temperatures with an accumulation of deformation into very narrow shear bands^8^. Lately, BMGs with additions of rare-earth elements showing high glass forming ability (GFA) and plasticity^9^,^10^ have been developed. These new-generation materials can meet the prospects of different sectors such as energy, safety, transportation, medicine etc. owing to their enhanced elastic and plastic behavior at room and elevated temperatures.

Recent investigations on the effect of long-term isothermal treatment of BMGs below the glass transition temperature conducted by different groups^11^,^12^,^13^ clearly identified the modifications of mechanical and thermal properties due to structural relaxation. This irreversible process is accounted for the annihilation of free volume via densification caused by annealing^8^. The decrease in free volume creates an additional endotherm on the differential scanning calorimetry (DSC) curve in the glass transition region without changing the glass transition temperature *T*_g_ or the exothermic enthalpy for crystallization Δ*H* of the BMG alloy. On the contrary, Van Steenberge *et al*.^14^ and Stoica *et al*.^15^ postulated the idea of low-temperature annealing through continuous heating of BMGs. This heating protocol increases the tendency for structural reordering of Cu and Zr atoms, and as a result, create considerable plasticity under compression. The concept behind the improved plasticity is linked to the short-term heat treatment at sub-*T*_g_ temperatures, where the structural modifications occur in the chemical and topological short-range order^12^. This mechanical property enhancement is achieved by continuous heating until the target temperature is reached, and rapid cooling of the sample immediately after the desired processing temperature is stabilized. By this method, the excess free volume within the BMG can be retained within the material. Subtle structural changes such as phase separation on the nano-scale or nucleation of nanocrystals distributed homogenously throughout the specimen account for the mechanical property enhancement of BMGs. Furthermore, metallic glasses can undergo *β* relaxation as low as 0.6 *T*_g_, which was shown to increase the macroscopic plasticity of BMGs through atomic diffusion^16^,^17^.

In particular, the mechanical and physical properties of BMG alloys are considerably influenced by the casting conditions and post-thermal treatment^18^,^19^,^20^,^21^. Although there have already been significant research done to explain the structural changes on the nano-scale upon such factors, the underlying mechanism for the mechanical property improvement is still a remaining question. Here, we present direct evidence for how the nanostructural events are related to create materials with enhanced resistance to softening via cross-examining the physical and (thermo)mechanical properties of CuZr-based BMGs with different thermal histories. In general, this study provides an effective approach to investigate the structural-property relationship in BMGs. Within this manuscript, we show the influence of thermal annealing at different temperatures and time intervals, and to what extent the mechanical and physical properties are altered by slight nanostructural modifications taking place in CuZr-based BMG.

## Results

**Figure 1:**
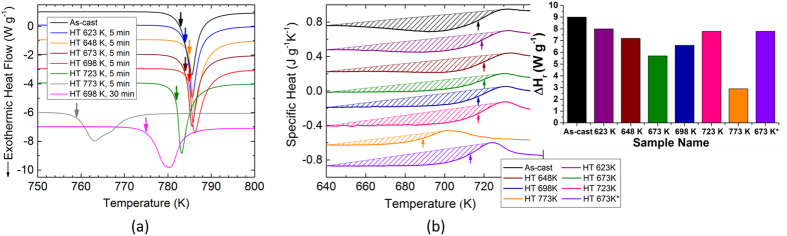
(**a**) DSC traces of as-cast reference and thermally treated samples at different processing conditions. The arrows indicate the onset of crystallization. (**b**) Change in specific heat as a function of temperature. The arrows indicate the glass transition temperatures for each thermal history. The inset shows the change in Δ*H*_r_ (which is calculated from the traces of Δ*c*_p_) at different annealing temperatures or times, where sample annealed at 673 K for 5 min shows the smallest value.

Cu_46_Zr_44_Al_8_Hf_2_ BMG was specifically developed for our current study, where minor additions of Hf (2 at.%) to the CuZr-based alloy were found to enhance the compressive plasticity and glass forming ability. The property improvement can be interlinked with the replacement of Zr with a heavier element with similar chemistry^22^,^23^. The annealing induced structural modifications on the course of heat treatment was investigated for various annealing temperatures as well as for different time scales. After annealing, the samples were subsequently cooled down to the room temperature at constant rates of 100 K/s to minimize the possibility of formation of undesirable phases (such as Cu_10_Zr_7_ and CuZr_2_)^21^. Figure 1 shows the continuous DSC curves of the samples that were previously isothermally annealed for 5 min at *T*_g_, as well as 25 K, 50 K, 75 K, and 100 K below the glass transition temperature. For comparison, the continuous heating curve of the reference (as-cast) state is presented. To understand the influence of above-*T*_g_ annealing and long-term sub-*T*_g_ annealing, a sample that was isothermally annealed for 5 min at 50 K above its glass transition, and a sample produced by annealing for half an hour at 50 K below *T*_g_ are presented, respectively.

The findings show that distinct relaxation mechanisms evolve at different temperatures. *T*_x_, the crystallization temperature, increases by several degrees (up to 2 K) after pre-annealing (Fig. 1a). On the other hand, *T*_g_, Δ*H* and the specific heat Δ*c*_p_ are marginally affected by the thermal history when the alloy is pre-annealed below its *T*_g_ for 5 min (Fig. 1b and Table 1). Significant *T*_g_ and *T*_x_ drops by 30 K and 25 K together with drops in Δ*c*_p_ and Δ*H* were observed for the sample treated at 773 K. Structural relaxation for half an hour below *T*_g_ decreases *T*_g_ and *T*_x_ by 5 K and 15 K, respectively, while not reducing Δ*H*. Interestingly and opposite to previous literature findings^11^,^24^, the enthalpy recovery in the calorimetric glass transition region Δ*H*_r_ was found to be smaller for the samples subjected to short-term annealing at sub-*T*_g_ temperatures (except for the HT 623 K sample) compared to the as-cast state sample and the sample annealed at *T*_g_ (723 K)(see Table 1 and Fig. 1b inset). This finding is possibly due to the inherited structural modifications (viz. phase decomposition, nanocrytallization etc.) during heat treatment^14^. Relatively higher temperature (or longer term) annealing creates an additional endotherm corresponding to an increased Δ*H*_r_, which finally decreases significantly for annealing above-*T*_g_. Thus, thermal analysis clearly shows that the thermal treatment of the HT 648 K, HT 673 K, and HT 698 K samples for very short durations has no major impact on changing *T*_g_, *T*_x_ and Δ*H* in contrast to annealing above *T*_g_. However, a remarkable drop in the Δ*H*_r_ values is observed for these three samples.

**Figure 2:**
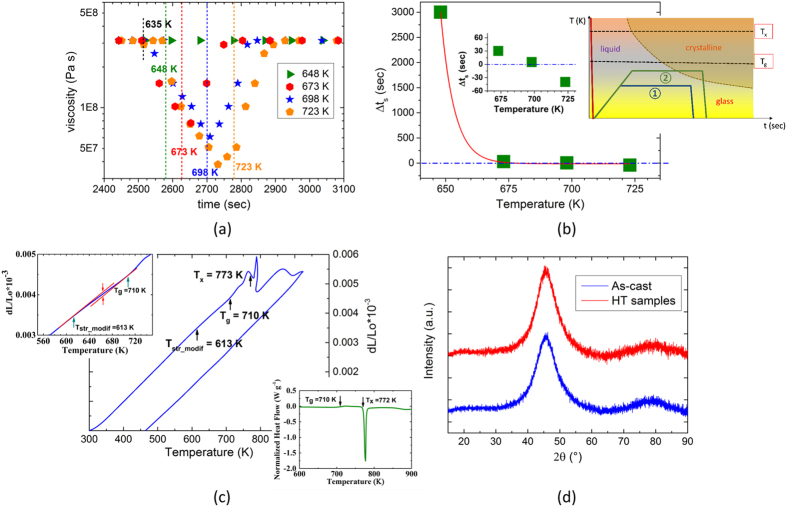
(**a**) Thermomechanical investigation of Cu_46_Zr_44_Al_8_Hf_2_. Viscosity as a function of time for isothermal heat treatments with different target temperatures below *T*_g_. The dashed lines indicate the point above which the samples reach to their isothermal temperature (*T*_iso_). (**b**) Crystallization time extracted from (**a**) [scale-up image is also given in the left inset]. *t*_*s*_ is the elapsed time to reach the destination temperature minus the corresponding time for the first scattered data point the viscosity start rising after the drop. The minus sign indicate that the onset of nanocrystallization initiated before reaching the target temperature. The exponential growth curve fit (red line) with an equation of *y* = −11.368 + 7.239 * 10^55^ * exp(−*x*/5.373) is selected (independent from the crystal nucleation and growth kinetics) to connect the data points for better understanding of the dramatic influence of process temperature on the structural characteristics. Time-temperature-transformation curve (right inset) illustrates different process routines of sub-*T*_g_ annealing. (**c**) Thermal expansion measurement by dilatometry. The scale-up image (upper inset) showing *T*_g_ and the onset point for structural modifications *T*_str_modif_ (arrows in dark cyan color), and the corresponding DSC curve at a constant heating rate of 10 K/min (lower inset). (**d**) Representative XRD pattern of the heat treated samples (at 623, 648, 673, 698, 723, and 773 K for 5 min, as well as 698 K for 30 min) compared to the as-cast samples, all of which show broad maxima.

Isothermal thermomechanical analysis (TMA) at sub-*T*_g_ (Fig. 2a) were conducted to investigate the relaxation kinetics of the selected BMG alloy as a function of the viscosity change. TMA can provide a highly sensitive mechanical analysis (displacement and temperature precision of 2 nm and ±1 K, respectively) so that even small fluctuations (caused by nanocrytallization, nanodefect formation, or nanosegregation) within the sample can be carefully traced. This measurement allows us to understand the connection between the thermomechanical and structural properties via the change in the viscosity value, and the influence of this change on the room-temperature mechanical behaviour.

The samples were heated to *T*_g_ of the CuZr-based BMG (*T*_g_ = 723 K measured by DSC) under vacuum, as well as to temperatures lower than the glass transition temperature (*T*_g_ − 25 K, *T*_g_ − 50 K, *T*_g_ − 75 K), respectively. The temperature was held constant until the viscosity rises and reaches to the thermal equilibrium again. The viscosity at a certain temperature as a function of time was calculated by applying the Stefan’s equation^25^,^26^:


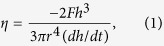


where *F* is the load applied by the plunger, *r* is the radius of the sample, and *h* is the height of the sample. The aspect ratio of the sample *h*/*r* was selected as 0.25 following^27^, and thereby the influence of the viscosity component of the liquid normal to the plates can be neglected.

The viscosity starts to drop around 635 K with increasing temperature. The change in viscosity differs with respect to the final temperature, where the isothermal TMA experiment at the glass transition temperature (723 K) shows an almost order of magnitude lower viscosity. Following this, it rises again and returns back to its original value which might be interlinked with the structural modifications (such as nanocrystallinity) within the structure.

**Figure 3:**
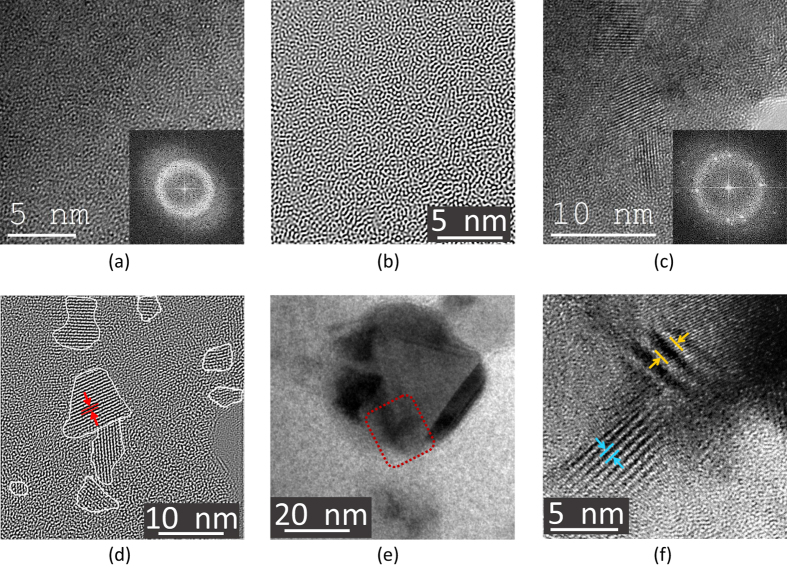
High-resolution TEM (**a,c**) and the corresponding inverse Fourier transformation (IFT) images (**b,d**) for the reference and HT 673 K samples. (**a,b**) A maze-like pattern is observed for the as-cast reference sample, where the fast fourier transformation (FFT) inset to (**a**) shows a broad diffuse halo. (**c,d**) The annealed sample shows nanocrystals of 2–5 nm (see the white encircled zones) indicated by the diffraction spots on the diffused halo obtained from FFT (**c** inset). The B2- CuZr crystals are identified from the lattice spacing of 0.326 nm. (**e**) Bright-field image of the HT 673 K sample displaying a nanocrystallite with dimensions of 50 nm and (**f**) the superpositioned lattice regions [area indicated by red dashed lines in (**e**)] due to the intersection of nanocrystallites at an angle to each other.

The dark yellow dashed line shows the first point where the temperature reaches to 723 K. The scattered data is indicated by dark yellow pentagons in Fig. 2a, where the minimum viscosity is reached at *T*_iso_*HT*723 *K*_ = 707 K. This clearly means that structural changes already starts to occur before the desired constant temperature is reached. As the isothermal temperature is lowered, the dashed line shifts to the left (i.e., shorter times), and the time until the material reaches the target temperature becomes comparable with the temperature at which structural changes start to occur. The blue dashed line in Fig. 2b and left inset indicate the point above which the samples reach their isothermal temperature (*T*_iso_). Green squares show the amount of available or overconsumed time (indicated by plus and minus signs, respectively) for the sample with respect to the blue dashed line. When the isothermal temperature is selected as 648 K or lower, no visible change in viscosity was observed for 30 min of waiting time. This temperature can be regarded as the threshold temperature below which no remarkable structural alterations are observed. However, this event might be due to the fact that the change in viscosity is overlapping with the device noise. For this reason, the minor decrease in the sample height for the mentioned sample was used to estimate the time of 3000 sec for the viscosity. The schematic time-temperature-transformation curve (right inset to Fig. 2b) shows the process path of the samples after casting (linear red line). The samples are subsequently annealed at sub-*T*_g_ for a short duration and do not intersect the crystallization curve (blue path) as opposed to the ones intersecting the curve with a possibility of crystallization (green path). The drop in viscosity followed by a subsequent rise in isothermal TMA measurements at sub-*T*_g_ temperatures show marked deviations of the viscosity data compared to the ones shown in the Angell plot^28^. Our results suggest that the stabilization of a nanocrystalline phase below *T*_g_ (see Fig. 3) causes this viscosity change.

Figure 2c shows the thermal expansion measurement of the CuZr BMG. *T*_g_ and *T*_x_ were found to be around 710 and 773 K, which is in good agreement with the data taken from the DSC curve conducted with the same heating rate (Fig. 2c bottom inset). The employed dilatometer has an ultra-high temperature precision of 0.1 K, and a displacement resolution Δ*l* of 8 nm. Hence, the structural modifications as a function of volumetric shrinkage can be identified during heating. The point where the curve deviates from linearity was selected as the temperature onset for the structural changes within the sample. The volumetric shrinkage, which is determined from the vertical difference between the two red dashed lines in Fig. 2c top inset, was calculated to be 3 × 10^−4^ (0.03%). This value is an order of magnitude smaller than the volumetric structural relaxation induced by the crystallization (0.5%), which is calculated from the vertical difference between the same heating and cooling dl/l_0_ data. XRD patterns obtained for the as-cast state, as well as for the samples with different thermal histories were compared to explore the influence of thermal treatment on the crystallization behaviour of BMGs. However, even after higher temperature or longer heat treatment, the samples showed broad patterns and no detectable diffraction peaks (Fig. 2d). This finding ascertains that the change in heat recovery can only be correlated with nano-scale structural modifications which cannot be identified by conventional XRD investigations.

Transmission electron microscopy (TEM) unraveled the change in the deformation pattern with respect to the annealing temperature. The as-cast reference sample’s corresponding high-resolution TEM (HR-TEM) image (Fig. 3a), and the inverse fourier transform (IFT) of the correlated images (Fig. 3b) revealed an amorphous maze-like pattern. On the other hand, the sample exposed to a heat treatment at 673 K for 5 min (HT 673 K) displayed nanocrystals which are below 50 nm in size that are dispersed homogenously (average of 20% by volume) within the amorphous matrix (Fig. 3c,d). The lattice constant from the place of local rearrangements was measured to be 0.326 nm corroborating the lattice spacing of the B2 CuZr phase found from X-ray diffraction^29^. In fact, B2-CuZr was previously found to primarily precipitate during sub-*T*_g_ annealing for long terms (on the order of hours)^24^. No compositional difference between the local ordered region and matrix could be detected by the energy dispersive x-ray analysis (EDX) in scanning transmission electron microscopy (STEM) mode. These findings confirmed that the phase precipitation is polymorphic^30^,^31^. Although the high temperature CuZr phase is normally stabilized above 700 K during fast quenching from the liquid state or during above-*T*_g_ heat treatment^21^, our findings suggest that the precipitation of the B2 CuZr nanocrytals are triggered by the high nucleation rates obtained at sub-*T*_g_ temperatures where the rate of atomic diffusion and growth of nanocrytals are limited^32^. Figure 3e depicts the bright-field image of a nanocrystallite with dimensions of around 50 nm formed by the same heat treatment. A remarkable contrast difference between different regions in the nanocrystals can be identified, which can be attributed to the superposition of lattice fringes from different B2 CuZr nanocrystals (as shown in Fig. 3f). The resultant patterns found on the nanocrystal precipitates within the glassy BMG matrix are named as moiré fringes^33^. The overall length of these fringes are measured to be around 5 nm, which indirectly suggests the grain size of these nanocrystals. The yellow and blue arrows show two different interference patterns with fringe spacings of 2.6 nm and 0.8 nm, respectively.

**Figure 4:**
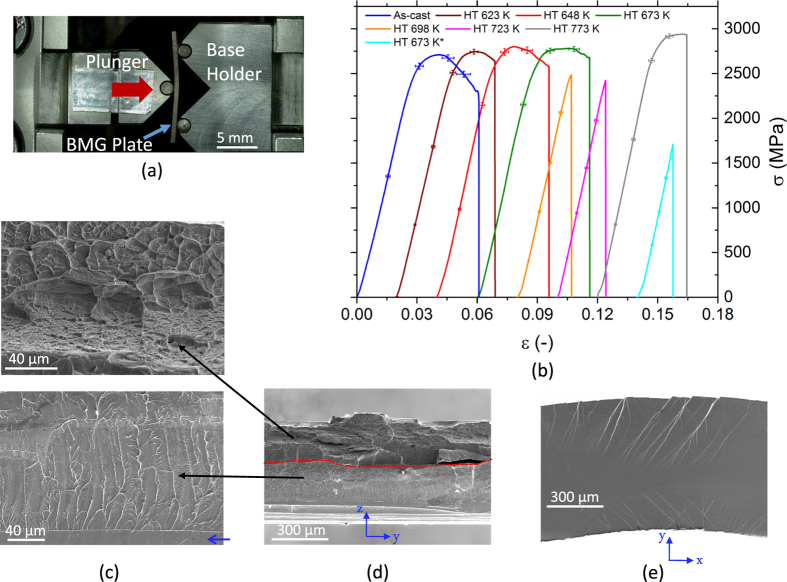
(**a**) The deformation setup for the bending test. The plunger was pushed at a strain rate of 0.001 mm/sec towards the fixed base holder. *In-situ* visualization of the experiment was carried by the VEDDAC video extensometer. (**b**) Stress-strain curves of the as-cast state and heat treated samples. Error bars are inserted for stress and strain values at equivalent data intervals for each sample. (**c**) Tensile (top) and compressive (bottom) sections of the bent specimen showing different fracture morphologies (yz axis). (**d**) Overall fractography of the broken sample. (**e**) Shear bands observed on the tensile and compressive sides of the shear surface (xy axis).

The current findings raise the question whether the nanocrystallization confirmed by the TEM study leads to changes in the mechanical properties on the macroscopic scale. For this purpose, by using the test setup shown in Fig. 4a, 3-point bending tests were performed. BMG samples at the selected thicknesses of 0.5 ± 0.05 mm are estimated to exhibit 5–6% bending plasticity^34^. The high extent of plasticity observed allows to determine the changes (i.e., amount of stress drop, plasticity, final strain at fracture) in the plastic regime with high accuracy and in a systematic manner. The findings revealed that the samples subjected to 5 min of heat treatment at 623, 648, 673 K followed by fast water quenching show a resistance to softening in the plastic regime in contrast to the as-cast reference sample (Fig. 4b). Thus, after reaching their ultimate flexural strength *σ*_UFS_, the strength drop for the heat treated (Δ*σ* = *σ*_UFS_ − *σ*_f_) samples became remarkably smaller (see Table 2). For the same samples, a higher elastic modulus *E* and *σ*_UFS_ was observed at only a slight expense of strain at rupture *ε*_f_. On the contrary, thermally treated samples 25 K below or at *T*_g_ showed a degraded *σ*_UFS_ with almost no plasticity. Particular attention should be given to the duration of the heat treatment. The HT 673 K sample with 5 min thermal treatment has higher *σ*_UFS_ and larger *ε*_f_ compared to the sample annealed for 30 min that shows catastrophic deformation below its elastic limit. High yield strength with limited plasticity was observed for the samples annealed above-*T*_g_ (HT 773 K), which can be attributed to the possible increase in the volumetric density of the crystals^21^.

The extensive bending plasticity observed for the as-cast state sample and the samples heat treated at 648 K and 673 K was reflected on the fracture surface of the specimens. The tensile side exhibits dimples which are created by the coalescence of microvoids during ductile fracture (Fig. 4c top figure). Vein patterns with partially molten ridges are the characteristics of the compressive side. The smooth region (pointed by a blue arrow) is regarded as being due to stable shear-type deformation before failure^35^,^36^ (Fig. 4c bottom figure). The difference in the fracture morphologies can be evidently seen in Fig. 4d. Sharp transition exists between uneven and smooth regions deformed under tension and compression, respectively. The extensive plastic strain for the mentioned samples is attributed to the amount of shear band formation. To show this correlation, another sample was deformed up to 80% of its average fracture strain, and the load was subsequently released (Fig. 4e). Sample analysis via high-resolution SEM reveal that the tensile side of the deformed specimen exhibits longer shear bands. Secondary and tertiary branching can be observed for the tensile side compared to shorter and less noticeable shear bands on the compressive side. The results are suggesting the precipitation of more nanocrystals on the tensile regions of the bent samples analogous to the stress-driven nanocrystallization process at room temperatures^37^. To conclude, the bend-tests of the 648 K and 673 K heat-treated samples show the highest resistance against softening which can be linked with the homogenous nanocrystal formation during thermal treatment in addition to the stress-induced nanocrystals generated during bending.

## Discussion

In this contribution, we developed a viable strategy to improve the overall toughness of BMGs by precipitating B2 CuZr nanocrystallites homogenously within the amorphous matrix through short durations of heat treatment at sub-*T*_g_ temperatures. The controlled structural modifications are reflected on the continuous DSC curves. The enthalpy recovery data in the calorimetric glass transition region Δ*H*_r_ are found to be decreasing for short terms of annealing at relatively lower annealing temperatures as opposed to the structures exposed to high temperature or long-term sub-*T*_g_ annealing. The isothermal TMA analysis displays a slight change in viscosity (approximately an order of magnitude). During the dilatometric measurement with continuous heating, the volumetric shrinkage measured between the initiation temperature of the structural modifications *T*_*str*_*modif*_ and *T*_g_ is found to be an order of magnitude smaller than the volumetric shrinkage ensuing between *T*_g_ and *T*_x_. These findings corroborate the slight structural modifications are taking place within the sample which happens before the growth of the nanocrystals, where in the latter case the glass is structurally relaxed by free volume annihilation. No devitrification of the matrix phase is detected by the (S)TEM measurements for the analyzed HT 673 K sample. The HT 673 K sample (*T*/*T*_g_ = 0.93) with homogenously dispersed nanocrystals (of sizes below 50 nanometers, and a volume fraction of ~20%) exhibit the highest resistance to softening with a maximum stress drop of only 3.6% (as opposed to the drop of 14.9% for the reference as-cast sample) with final strain values comparable to the as-cast state sample. The contrast difference between different regions in the nanocrystals is observed in the HT 673 K sample, where the detected lattice fringes are due to the overlapping of different B2 CuZr nanocrystals which form during the heat treatment process.

In summary, the thermal stability of the BMG changes with the chosen annealing temperature and time. Intrinsic property amelioration can be achieved by the controlled and homogenous nanocrystallization proposed within this study. We envision that the presented method can enable us to design tough metallic glasses with tailorable properties.

## Methods

The BMG master alloy ingot was prepared from elements with purity higher than 99.99% using an Edmund Bühler GmbH Arc Melter. The master alloys were subsequently heated above the liquidus temperature three times to form a homogenous mixture. The master alloys were then sliced into 10–12 g pieces, and the water-cooled copper mold casting was conducted under Ar atmosphere using an *in-situ* suction casting device attached to Edmund Bühler Arc Melter. The dimensions of the cast plates were 1.5 mm × 10 mm × 75 mm. The cast plates were sliced into smaller pieces (using Struers Accutom 50) and slightly grinded by hand to eliminate surface oxides. The plates were screened by 3D computer tomography (GE Phoenix Nanotom), and no porosity on the micron-level was found. The sample plates were inserted in a calibrated furnace, and brought to different temperatures below *T*_g_ under Ar atmosphere of 10^−5^ mbar. After reaching the chosen temperature, samples were isothermally treated for 5 min for temperature stabilization, and subsequently taken out of the furnace and water-quenched to room temperature. The as-cast reference and heat treated samples were grinded and subsequently mirror-polished down to the desired thickness level using Buehler Metaserv 250 to eliminate any influence of possible surface crystallization and oxidation. The final dimensions of the samples had a thickness of 0.5 ± 0.05 mm, where the width and the length of the samples were 4.5 ± 0.3 mm and 14.0 ± 1.0 mm, respectively. Meanwhile, smaller pieces (about 20 mg in weight) were prepared for calorimetric measurements. Using a PerkinElmer Pyris Diamond DSC, each piece was heated to its destination temperature at a rate of 20 K/min, kept at constant temperature for 5 min, and finally cooled down at a rate of 100 K/min. The glass transition (*T*_g_) was measured from the inflection point of the endothermic ramp, whereas the crystallization temperature (*T*_x_) was measured from the onset point of the exothermic signal. Viscosity measurements were conducted with a Perkin-Elmer Dynamic Mechanical Analyzer (DMA 7) with 3 mm diameter flat tip parallel plate probes. The samples (2 samples from each set) were heated up to the desired temperatures at a heating rate of 20 K/min under a constant load of 2.6 N, and were subsequently annealed isothermally for 0.5 h followed by fast cooling of 100 K/s. The relative length change dl/l_0_ of the Cu_46_Zr_44_Al_8_Hf_2_ samples was measured using a Netzsch DIL 402 C Dilatometer under a constant heating and cooling rate of 10 K/min with two identical samples. The length of the samples were 27.96 ± 0.12 mm with a diameter of 3 mm, where both sides were made parallel by mirror polishing. Structural characterization was conducted before and after heat treatment by X-ray diffraction using a D3290 PANalytical X’pert PRO with Co-K*α* radiation. For the mechanical property characterization, 3-point bending tests were performed with the Kammrath & Weiss Tensile/Compression Module (with 5 kN load cell) with attached bending fixtures having an effective gauge section of 10 mm. Samples with thermal histories, as well the as-cast reference samples were deformed under strain rates of 10^−3^ 1/s until rupture. The imaging with the preloaded state is conducted by Keyence Digital Microscope VHX 2000. The visualization of the deformation process, and the strain correction was implemented using a video extensometer system (MicroDAC strain measurement) attached to the testing device. The fractographic analysis after rupture, as well as shear surface analysis of the bent samples right before failure were performed using an SEM Zeiss Ultra Plus and its EDX detector attached. The fractographic analysis were also conducted for different samples of *ε*_f_ > 5.0%, however because of the similar deformation patterns and final strains, the images only from the as-cast sample were used to represent the other samples of interest. The specimens for the TEM observations were prepared by ion milling (Gatan 691) via liquid nitrogen cooling. The amorphous structure and nanocrystallinity of the as-cast and heat treated samples were investigated using a TEM FEI Tecnai F30. The composition was analyzed by an EDX module attached to the TEM.

## Additional Information

**How to cite this article**: Sarac, B. *et al*. Towards the Better: Intrinsic Property Amelioration in Bulk Metallic Glasses. *Sci. Rep*. **6**, 27271; doi: 10.1038/srep27271 (2016).

## Figures and Tables

**Table 1:** DSC data for samples with different thermal histories at a heating rate of 20 K/min, followed by short-term annealing after reaching their target temperature.

Sample	*T*_*g*_ (±2 *K*)	Δ*c*_*p*_ (±0.01 *Jg*^−1^*K*^−1^)	*T*_*x*_ (±2 *K*)	Δ*H* (±0.7 *Wg*^−1^)	Δ*H_r_* (±1.0 *Wg*^−1^)
As-cast	723	0.26	783	−54.8	9.0
HT 623 K	719	0.25	784	−54.4	8.0
HT 648 K	720	0.23	785	−54.6	7.2
HT 673 K	720	0.20	784	−54.3	5.7
HT 698 K	717	0.28	785	−54.7	6.6
HT 723 K	717	0.22	782	−54.9	7.8
HT 773 K	689	0.17	759	−48.4	2.9
HT 673*K	713	0.27	775	−53.8	7.8

Note that the highest standard deviation values among the samples were taken into account.

^*^Held for 30 min at constant temperature.

**Table 2:** Mechanical properties extracted from the 3-point bending test.

Sample	*T*/*Tg*	*E* (±1 *GPa*)	*σUFS* (±50 *MPa*)	*σ_f_* (±50 *MPa*)	Δ*σ* (±1.2%)	*ε_f_* (±0.1)
As-cast	–	97	2709	2305	14.9	6.1
HT 623 K	0.86	98	2745	2639	3.9	4.9
HT 648 K	0.90	104	2816	2593	7.9	5.6
HT 673 K	0.93	103	2778	2679	3.6	5.6
HT 698 K	0.97	103	2535	2535	–	2.7
HT 723 K	1.00	105	2447	2447	–	2.4
HT 773 K	1.07	106	2942	2933	–	4.4
HT 673^*^ K	0.93	106	1729	1729	–	1.8

The term *T*/*T*_g_ defines the normalized annealing temperature with respect to *T*_g_ of the as-cast BMG. *σ*_f_ is the fracture strength of the bent specimens. Δσ is the ratio of strength drop between *σ*_UFS_ and *σ*_f_. Note that the highest standard deviation values among the samples were taken into account.
